# Transposable-Element Associated Small RNAs in *Bombyx mori* Genome

**DOI:** 10.1371/journal.pone.0036599

**Published:** 2012-05-08

**Authors:** Yimei Cai, Qing Zhou, Caixia Yu, Xumin Wang, Songnian Hu, Jun Yu, Xiaomin Yu

**Affiliations:** 1 CAS Key Laboratory of Genome Sciences and Information, Beijing Institute of Genomics, Chinese Academy of Sciences, Beijing, China; 2 Laboratory of Biochemistry and Molecular Biology, National Cancer Institute, National Institutes of Health, Bethesda, Maryland, United States of America; Georgia Institute of Technology, United States of America

## Abstract

Small RNAs are a group of regulatory RNA molecules that control gene expression at transcriptional or post-transcriptional levels among eukaryotes. The silkworm, *Bombyx mori L.*, genome harbors abundant repetitive sequences derived from families of retrotransposons and transposons, which together constitute almost half of the genome space and provide ample resource for biogenesis of the three major small RNA families. We systematically discovered transposable-element (TE)-associated small RNAs in *B. mori* genome based on a deep RNA-sequencing strategy and the effort yielded 182, 788 and 4,990 TE-associated small RNAs in the miRNA, siRNA and piRNA species, respectively. Our analysis suggested that the three small RNA species preferentially associate with different TEs to create sequence and functional diversity, and we also show evidence that a *Bombyx* non-LTR retrotransposon, bm1645, alone contributes to the generation of TE-associated small RNAs in a very significant way. The fact that bm1645-associated small RNAs partially overlap with each other implies a possibility that this element may be modulated by different mechanisms to generate different products with diverse functions. Taken together, these discoveries expand the small RNA pool in *B. mori* genome and lead to new knowledge on the diversity and functional significance of TE-associated small RNAs.

## Introduction

Transposable elements (TEs) usually make up a substantial portion of eukaryotic genomes and play evolutionarily unique roles in maintaining diversity, integrity and stability of the genomes. According to the mode of propagation, TEs are generally classified into DNA transposons that move through a ‘cut-and-paste’ mechanism and retrotransposons (including SINE, LINE and LTR) that develop more complicated mechanisms in proliferation [1]. Among insect genomes, the repeat content varies greatly: 1% in *Apis mellifera* [2]; 16% in *Anopheles gambiae* [3]; 33% in *Tribolium castaneum* [4], and 47% in *Aedes aegypti* [5]. Even within the same genus, the twelve sequenced *Drosophila* genomes have repeat contents from 2.7% to 25% [6]. The repetitive sequences in *Bombyx mori* are estimated to be 43.6% [7], [8]. TEs have been shaping insect genomes by manipulating sequence (genetic) content and diversity through their expansion and promoting rearrangement [9].

Small non-coding RNAs are a group of short RNA molecules that silence a wide range of genes transcriptionally and post-transcriptionally. The group composed of three major classes of small RNAs: microRNAs (miRNAs; 21–24 nt), small interfering RNAs (siRNAs; 21–22 nt) and Piwi-interacting RNAs (piRNAs; 24–30 nt) [10]. Usually, miRNAs originate from stem-loop structures transcribed from the non-coding region of genomes. However, increasing lines of evidence suggest that when insert into actively transcribed regions, some TEs generate functional miRNAs and this phenomenon may serve as one of the evolutionary mechanisms for miRNA formation [11], [12], [13]. In addition, it was reported that about 20% of known human miRNAs are derived from repetitive elements [14], [15]. Endo-siRNAs (esiRNAs), the major performer of RNAi, have been widely described in plant, fission yeast, nematode [16], and recently fly [17], [18], [19], [20] and mouse [21]. These esiRNAs are concentrated in soma and are considered as a protecting mechanism against transposons, much as piRNAs do in the germ line. piRNAs are regarded as a conserved mechanism responsible for the genomic integrity and stability in germ cells, preventing insertional mutagenesis caused by TEs and protecting the DNA from double-stranded breaks [22]. There is evidence that piRNAs play key roles in the developmental regulation of fly germ lines [23], [24], [25]. Generally, piRNAs can be divided into two groups, primary piRNAs and secondary piRNAs. In flies, primary piRNAs come from maternal deposition or the processing of some special loci (such as *flamenco* and *traffic jam*) and the 3′ UTR of an extensive set of mRNAs [26], [27], [28], [29], whereas the genesis of secondary piRNAs undergoes ‘Ping-Pong Circle’. In this model, piRNAs operate in an amplification loop in which transposon sense transcripts trigger the production of antisense piRNAs and transposon antisense transcripts induce the generation of sense piRNAs [28].

For the past few years, Kawaoka and co-workers have made great efforts in the study of small RNAs, especially piRNAs that control transposons in *B. mori* germ line cells. First, they cloned thousands of *Bombyx* small RNAs from pupal ovaries that associated with transposons or repetitive sequences and classified them as repeat-associated small interfering RNAs (rasiRNAs), a subclass of piRNAs [30]. Second, they identified two PIWI subfamily proteins, silkworm Piwi (Siwi) and Ago3 (BmAgo3), and associated piRNAs with conventional signatures in a ovary-derived cell line, BmN4 [31]. These results offer a molecular basis for the biogenesis and function of piRNAs and indicate that the piRNA amplification loop proposed in *Drosophila* is evolutionarily conserved in *Bombyx*. Third, by large-scale profiling of piRNAs from silkworm ovary and embryos of different developmental stages, they demonstrate that maternally inherited antisense-biased piRNAs can trigger acute amplification of secondary sense piRNA production in zygotes, at a time coinciding with zygotic transcription of sense transposon mRNAs, which provide a proof for the ‘Ping-Pong Circle’ mechanism [32]. Besides, they also provided experimental evidence for the biosynthesis mechanism of piRNAs using BmN4 cell line [33].

We previously constructed a small non-coding RNA library of *B. mori* and deeply sampled it using the ABI SOLiD platform. Our analysis discovered 287 new miRNAs including conserved and species-specific ones [34]. In favor of the notion that a non-negligible subset of small RNAs are derived from TEs and are broadly related with developmental regulation and other biological processes [11], [23], [35], [36], we further scrutinized the sequencing data to identify TE-associated small RNAs as well as their potential functions in *B. mori* genome.

## Results and Discussion

### TE-associated miRNAs

Nearly half of the silkworm genome is occupied by TEs [8]. Given the mechanisms of small RNA biogenesis, it is conceivable that actively transcribed TEs provide an opportunity for generating small non-coding RNAs. We mapped known silkworm miRNAs (miRBase16.0) and refined reads in a length range of 21–24 nt to the silkworm TE database. After computational screening and manual inspection, we identified 201 (representing 182 TE-miRNAs) miRNA precursors structurally derived from TEs, and 22 of them are previously reported miRNAs (Table S1). The TE-derived miRNAs we identified are from all four major TE types: LINE, SINE, LTR and DNA transposons (the bulk belongs to LINE). The base composition of TE-miRNAs does not show 5′ U preference as compared to non TE-miRNAs [34]. In addition, we also predicted 160 new TE-miRNAs including ten that have both miRNA and miRNA* strand detected (Table S1).

We observed a similar mechanism in silkworm TE-miRNAs, as we previously reported in non TE-derived miRNAs [37], that a miRNA precursor is able to yield different kinds of mature miRNA sequences (such as TE-miRNA-1 and TE-miRNA-2), and likewise different precursors produce the same miRNAs. In addition, the pairing relationship between miRNA and miRNA* is also not strict. A mature miRNA may match to more miRNA*s and *vice versa*. As reported, 5′-end recognition by Dicer is important for precise and effective biogenesis and functionality of miRNAs [38]. However, the 3′-end is more flexible usually with 1–3 nt overhang and when the length of the overhang increases from 1 to 3 nt, the position of the preferred Dicer cleavage site shifts [39]. Hence, the inaccuracy of Dicer processing may lead to accumulation of the precisely processed strand with a conserved 5′-end. If this bias is true for miRNA biogenesis, it offers a good opportunity to understand the functional importance of the two miRNA strands [20], [40], [41].

Sequence comparisons revealed that TE-derived miRNAs are less conserved (except for miR-1923, miR-3314 and miR-3318) than non-TE-derived miRNAs, as in the case of human TE-miRNAs [11]. Those TE-derived miRNAs are highly specific, inconsistent with the discovery of Smalheiser and Torvik [12], but in concert with the findings by Piriyapongsa *et al* [11]. Smalheiser and Torvik found several mammalian miRNAs that derive from TEs are highly conserved among human, mouse and rat. Piriyapongsa and colleagues gained 140 human TE-miRNA genes with the potential to regulate thousands of human genes. Sequence comparisons revealed that those human TE-derived miRNAs are less conserved, on average, than non-TE-derived miRNAs, which echoed our discovery. Hence, the conservation of TE-miRNAs as well as the evolutionary relationship between TE-derived miRNAs and non TE-derived miRNAs is hard to determine due to a limited availability of the data. Target prediction did not show obvious differences between TE-derived miRNAs and non TE-derived miRNAs. Those potential targets are involved in a broad range of biological functions, such as gene transcription and translation, signal transduction, metabolism and so on (Table S2).

### TE-associated siRNAs

**Figure 1:**
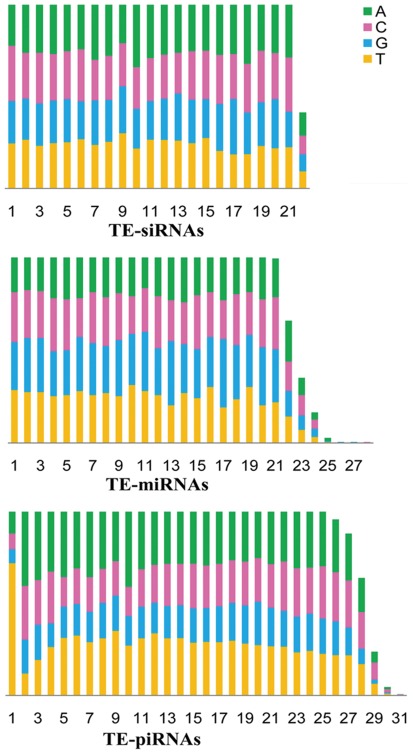
Base composition of TE-associated small RNAs. This histogram depicts the base distribution of TE-associated small RNAs in the three classes, whose length ranges are 21–27 nt, 21–22 nt and 25–31 nt for miRNAs, siRNAs and piRNAs, respectively.

For a long time, siRNAs are thought to generate from exogenous dsRNAs to perform RNA interference. Endo-siRNAs come to light only recently and a bunch of endogenous double-stranded RNA substrates are regarded as their biogenesis resources including long hairpin structures [20], overlapping transcription units [21], [42] and transposable elements [17], [19]. TEs are proven to be one of the main resources for the genesis of endo-siRNAs in fly [17], [18], [19]. The TE derived endo-siRNAs (TE-siRNAs), functioning in a piRNA way, built a solid defending system against transposable elements in soma [43]. In an effort to identify TE-derived siRNAs in *B. mori*, a subset of qualified reads with a length distribution between 21–22 nt is subjected to be probed for TE-siRNAs after subtracting the candidate TE-miRNAs. We gathered 788 candidates, 19.3% of which are mapped to multiple locations (Table S3). The frequency of those candidates span a broad range from a few tens to a few thousands with 62.8% sequenced more than 20 times. The base preference of the candidate TE-siRNAs revealed a slight C-rich trend in the 5′ end (Fig. 1), which is similar to endo-siRNA populations from *Drosophila* [18]. Curiously, 60% TE-siRNAs were matched to the antisense strand of a silkworm TE (bm1645) that belongs to R4 clade (discussed later). This antisense bias observed in the TE-siRNA candidates is inconsistent with exo-siRNAs and previous reports with unknown reason [10], [18].

### TE-associated piRNAs

Because piRNAs are generated mainly from germline cells, the percentage of piRNAs annotated in our small RNA library which is constructed from the whole body of silkworms is limited (4.8% of mappable reads). After a series of screening, we obtained 4,990 TE-piRNA candidates, 68.2% of which are matched to a single site and about 32.4% candidates have no less than 20 copies (Table S3). In our dataset, 600 (∼12.0%) candidate TE-piRNAs are also experimentally cloned from *Bombyx* ovary [30]. Most of the *Bombyx* TEs generated piRNAs from both sense and antisense strands either along the entire gene or focusing on certain hotspots. TE is the major source for the birth of piRNAs but not the only one [31]. Given the less conservation of piRNAs and the fact that some heterochromatic regions and other repeat sequences can also give birth to piRNAs, our results only represent a small subset of piRNA molecules in silkworm, which still exhibit typical features of piRNAs. The base distribution of the silkworm TE-piRNAs has distinct 5′ U and 10A bias, which are canonical features of piRNA sequences [10]. Among the uniquely mapped TE-piRNAs, approximately 58% candidates are derived from antisense strand of TEs, another characteristic of strand preference of piRNAs. The top 20 TE-piRNA clusters as well as their mapping reads are summarized in Table 1. A remarkable piRNAs bias is noticeable (also see Figure S1)–a high fraction of antisense piRNA produced from TEs, including bm1645, Moriya and HOPEBm2, although certain TEs are mapped dominantly as sense piRNAs (bm679, bm1695, Kagayaki, bm1266, bm1087, and bm939). This result is partly accordant with what is found in mouse–TE-piRNAs and TE-siRNAs preferentially related to different TEs [21], [42].

**Table 1:** Small RNAs mapped to the top 20 TE-piRNA clusters.

TEs	Type	piRNAs		siRNAs	
		Sense	Antisense	Sense	Antisense
bm1645	LINE/R4	0	138(36,711)	0	476(107,799)
Moriya	LTR/Pao	9(115)	82(5,159)	5(56)	3(53)
bm1796	LTR/Gypsy	11(89)	64(1,162)	1(5)	7(94)
bm679	DNA/P	46(11,131)	22(534)	2(20)	2(231)
bm1695	DNA/piggybac	42(4,025)	17(657)	0	4(144)
bm939	LINE/L2	52(2,341)	6(187)	0	4(76)
Orochi	LTR/Pao	20(221)	37(609)	0	0
bm1087	LINE/I	40(1,246)	16(187)	0	1(5)
HOPEBm2	non-LTR	2(11)	51(1,482)	0	2(40)
bm789	LTR/Gypsy	4(42)	45(995)	0	0
TREST-W	non-LTR	7(87)	41(1,214)	2(16)	6(59)
Pakurin	non-LTR	6(43)	38(1,277)	0	1(8)
bm1266	SINE/SINE	33(2,026)	8(173)	0	0
Kagayaki	non-LTR	31(1,239)	9(137)	1(8)	0
Kimono	LTR/Pao	2(20)	38(757)	1(5)	0
Taguchi	non-LTR	2(16)	37(992)	0	2(35)
bm1052	LTR/Gypsy	1(13)	37(1,036)	0	0
bm1815	DNA/Harbinger	11(761)	27(1,178)	0	0
Nemawashi	LTR	1(13)	35(1,205)	1(6)	1(54)
BmpiggyBac-MER85	DNA	3(324)	31(4,497)	0	0

* Total reads of candidate TE-siRNAs and TE-piRNAs are shown in parenthesis.

During the preparation of this manuscript, Kawaoka *et al* published a new set of piRNAs from gonads of silkworms which is a valuable resource for comparative analysis. Therefore, we compared our TE-piRNA candidates to the piRNAs from ovary and testis of wild type silkworm p50T. As a result, our data has 58.8% and 48.3% overlap (no more than one mismatch) with ovary-derived piRNAs and testis-derived piRNAs, respectively. These results partially reflect the reliability of our dataset. Furthermore, we compared our data with the sequences generated by SIWI/BmAGO3 immunoprecipitation method. The overlap (zero mismatch detected) between our data and BmN4-derived piRNAs, Siwi-bound piRNAs, and BmAgo3-bound piRNAs are 13.2%, 16.3% and 35.7%, respectively. Attempts to increase matching rate by loosening the threshold to three mismatches was not productive. The limited overlapping rates are expected since the reasons are multifold. For instance, our data were generated from a library made from RNA of entire silkworms and the background differences between silkworm sample and cultured cell line. Other factors include differences in experimental protocols and data processing strategies.

According to the piRNA ‘Ping-Pong Circle’, antisense TEs generate antisense piRNAs that load AUB (or PIWI), which induce the cleavage of active TE transcripts and result in the birth of sense piRNAs that load AGO3 [10]. Therefore, this mechanism defines a piRNA pair that is complementary by 10 nt, for which the guide strand begins with U (5′ U-piRNAs) and its target strand has an A at position 10 (10A-piRNA). In our library, we meticulously singled out 539 piRNA pairs satisfying above conditions, which are probably yielded from the ‘Ping-Pong Circle’ and make up 18% of all TE-piRNAs. The percentage of the 10A-piRNAs from the sense strand and 5′ U-piRNAs from the antisense strand are 43% and 57%, respectively. Since piRNA pairing is only restricted by the 5′-end 10 nt sequence overlap, there is no surprise for us to observe that one 5′ U-piRNA may direct the production of more than one 10A-piRNAs and *vice versa*. In other words, the 5′-end sequence of TE-piRNAs produced through ‘Ping-Pong Circle’ is more conserved than their 3′-end sequence. This phenomenon supports in part the idea that piRNAs are highly diverse and less conserved even among closely related species [44].

### Bm1645: A Pool of Small RNAs

**Figure 2:**
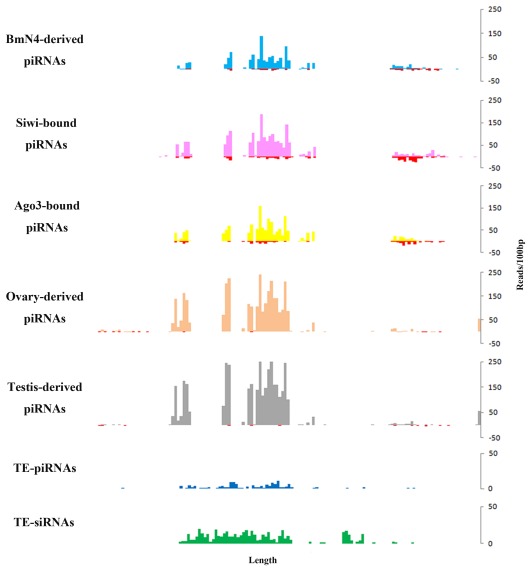
The expression pattern of bm1645. piRNAs from different datasets are displayed in a 100 nt sequential window and those mapping to the antisense strand of bm1645 are pictured in different colors and those mapping to the sense strand are indicated in red.

It is reported that not only a single transcript may become a substrate for the biogenesis of both piRNA and siRNA but also distinct classes of transcripts can arise from a single locus [18]. In the process of predicting TE-associated small RNAs, we identified a large TE, bm1645, which has the potential to generate three classes of small RNAs. To further demonstrate this, we folded the small RNA concentrated region of the antisense strand of bm1645 at a temperature of 30 centigrade [45] and received a potential structure for the biogenesis of small RNAs that initiated from dsRNA regions (including hairpins, see Figure S2). We also mapped silkworm gonad-derived, BmN4-derived and SIWI/BmAGO3-bound piRNAs to bm1645. As shown in Fig. 2, piRNAs enriched from gonads/BmN4 also display a pronounced antisense bias, and the result supports the observation of the extreme antisense bias in our data (no reads mapped to the sense strand of bm1645). In addition, the expression pattern is quite consistent among different datasets except for a few fragments and such fact implies the reliability and functional significance of bm1645 related small RNAs. This overwhelmingly antisense preference of bm1645 for generating small RNAs also suggests an involvement of strand-biased expression regulation.

**Figure 3:**
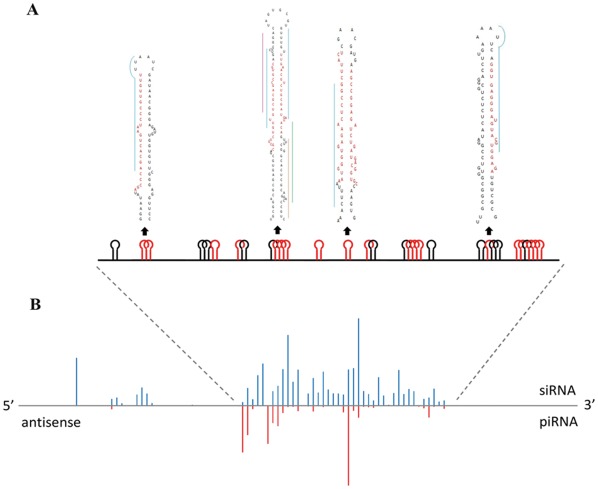
TE-associated small RNAs mapped to bm1645. TE-miRNAs structurally originated from hotspot regions of the bm1645 antisense strand are shown in a 5′–3′ orientation, where a large number of TE-siRNAs and piRNAs are also concentrated (A). Representative hairpins with more than one mature miRNAs/miRNA*s are highlighted in different colors (red). The read density of TE-associated siRNAs (blue) and piRNAs (red) matching to the antisense strand of bm1645 are displayed (B). Only uniquely mapped sequences are accounted for computing the read density in a 100 nt sequential window.

A majority of TE-miRNAs (73%) is originated from the antisense strand of bm1645. The details of the candidate TE-miRNAs as well as their relative mapping locations are described in Fig. 3A. It is striking that some parts of bm1645 are polytropic in forming different types of miRNA precursors which are mixed up but also discriminate from each other regarding to mapped reads. Comparative analysis suggests that this transposon is less homologous or has limited homology to other insect TEs. As far as TE-piRNAs are concern, bm1645 represents the biggest cluster and produces 138 candidates (Table 1). We counted the mapped reads to candidate TE-siRNAs and TE-piRNAs in a 100-bp window (Fig. 3B) and the small RNAs are concentrated on a 6.3-kb to 10.4-kb region of the antisense strand, which is also the hotspot for TE-miRNAs.

It seems that the chance in generating TE-siRNAs or TE-piRNAs is not equal or random for bm1645. In other words, some fragments tend to produce more piRNAs than siRNAs or *vice versa*. This idea is also supported by other TEs listed in Figure S1. To further confirm this, we downloaded small RNAs from a ovary somatic sheet (OSS) cell line from fruit fly [10] and processed the sequencing reads in our pipelines. The result is highly consistent with our conclusion that piRNA/siRNA prefer to control different TEs and a TE gene itself may be regulated by different mechanisms to generate different kinds of small RNAs (Figure S3). Although it has been reported that some piRNA clusters are able to generate endo-siRNAs in flies and mice [17], [43], no significant connections of these two pathways was observed due to two facts. First, piRNA pathway mutations have little impact on siRNA populations and siRNA pathway mutations do not affect piRNA pools [17], [28], [35]. Second, the same probes that detected TE-siRNAs in S2 cells hybridized to TE-piRNAs in female bodies in fly [43]. Therefore, this notable unbalance indicates that bm1645 may be regulated by different mechanisms rather than a random process and may yield different kinds of small RNAs. As a caveat, it would be valuable and necessary to further analyze the functional importance of bm1645.

### Conclusion

The silkworm genome is abundant in transposons, which made up a great part of the genome and provide potential resources for the biogenesis of small RNAs. In this study, we focused on a subset of TE-associated small RNAs that affect the accumulation of a large number of TEs. The small RNAs discovered herein only represent the tip of the iceberg and are less conserved between species or among different datasets due to the diversity nature of the biogenesis mechanisms as well as many other technical reasons which together bring great challenges in sequence comparisons based on homology, especially when the sequences are actually very short. However, a majority of TE derived small RNAs also displayed remarkable characteristics and can be considered as latent regulators of corresponding transposons. Our analysis on TE-associated small RNA lead to a conclusion that different TEs may be controlled by different small RNAs and even a single TE gene is capable of generating different products and may be regulated by different mechanisms.

## Materials and Methods

Silkworm samples used in this study were previously depicted in reference [37]. In short, we collected silkworms at 14 different developmental stages from eggs to moths and total RNA was extracted separately by TriPure Isolation Reagent (Roche) according to manufacturer’s protocol. Those total RNAs were mixed equally and small RNA fragments in a length range of 18–40 nt were recovered for constructing high-throughput sequencing library. The sequencing reads were generated from SOLiD platform (also see reference [34] for details). After screening out annotated non-coding small RNAs, mRNAs and miRNAs from the dataset to avoid interference from degraded transcripts and other contaminations, the remaining reads were refined and sent into a well-designed analysis pipeline to define relationship between small RNAs and TEs. We processed the reads mapped to silkworm genome but not annotated by clustering the redundant reads into unique groups and collected those in a size range of 21–31 nt and no less than 5 in frequency. Since it is hard to find out genuine small non-coding RNAs from a library made from samples of different developmental stages, we used discreet strategies for the identification of different small RNA categories.

Databases downloaded for bioinformatics analysis are listed as follows: miRBase 16.0 (http://www.mirbase.org/); 105 well annotated silkworm TE sequences are obtained by searching NCBI nucleotide database (http://www.ncbi.nlm.nih.gov/nuccore) according to references [31], [32]; BmTELib dataset is gained from Silkworm Genomic Research Program (http://sgp.dna.affrc.go.jp/pubdata/genomicsequences.html) and only annotated sequences are used. Silkworm unigenes were extracted from NCBI for miRNA target prediction. Silkworm piRNA databases used for comparative analysis were retrieved from NCBI and DDBJ (ftp://ftp.ddbj.nig.ac.jp/ddbj_database/) according to their accession numbers [30], [31], [32]. piRNAs generated from ovary and testis of p50T were obtained from DDBJ under accession number DRR000433 and DRR0000434, respectively [46]. The small RNAs from *Drosophila* ovary somatic sheet (OSS) cell line were downloaded from GEO dataset under accession number GSM385744 [10]. *Drosophila* TE database used for small RNA analysis was downloaded from FlyBase (http://flybase.org/static_pages/lists/dmel_te.html).

We mapped known silkworm miRNAs (miRBase16.0) and refined 21–24 nt reads to the silkworm TE database. Short reads mapped perfectly to the reference were extracted together with 100 nt flanking sequences for hairpin structure prediction by using Mfold (G:U wobble is tolerated) [47]. After step-by-step screening, structures fit the following conditions are considered as candidate miRNA precursors: 1) the minimum free energy of the hairpin is ≤−20 kcal/mol; 2) reads have ≤4 continuous mismatches; and 3) most reads locate in the stem and have at least 16 nt base-pairing to the complementary strand. Next we sought to find if there were miRNA* strand for the candidate TE-miRNAs in our library, which is also located in the stem-loop structure of miRNA precursor and complementary to the mature miRNA. We searched the remaining reads to the predicted hairpins and picked those reads that perfectly matched to the hairpin structure and complementary to mature miRNA with ≤2 nt 3′ overhang as miRNA* strand temporarily [39]. TE-miRNAs’ target prediction was performed by miRanda (v3.1) [48] under parameters of -sc 140, -en −20.

We selected 21–22 nt reads for probing TE-siRNAs [20], [21]. After filtered out reads predicted to be TE-miRNAs in the previous step, the remaining set of reads are mapped to the TE database again. Using in-house developed Perl scripts combined with manual inspection, we chose reads perfectly complementary to the reference. The processed reads with length distribution between 25 nt and 31 nt were also aligned to the silkworm TE database and were analyzed by Perl scripts for the identification of candidate TE-piRNAs. No mismatch was allowed and only uniquely mapped reads were used for the cluster analysis because the location of multiply-matched reads is undistinguishable. Sequences containing ≥20 short reads within 20 kb were considered as TE-piRNA clusters [49]. To confirm piRNA/siRNA prefer to control different TEs, we mapped fly siRNAs and piRNAs to fly transposable elements by the same pipeline.

For the study of bm1645, we mapped reads from p50T ovary and testis, BmN4-derived piRNAs, Siwi-bound piRNAs, and BmAgo3-bound piRNAs, to the antisense strand of bm1645 with no mismatch. The same database constructed for filtering annotated mRNAs, rRNAs, tRNAs and other non-coding RNAs was also used for filtering the bm1645 mapped reads by using SOAPaligner/soap2 [50]. And only reads with length distribution between 25–31 nt and mapping to unique positions were kept for further analysis.

## Supporting Information

Figure S1 **Representative silkworm TEs with mapped small RNAs.** Silkworm TE-associated piRNAs and miRNAs are shown in red and blue, respectively.
(PDF)

Figure S2 **RNA folding results of the antisense strand of bm1645.**
(PDF)

Figure S3 **Fly TE mapped small RNAs.** (A) TEs with small RNA generating bias. This part shows representative TEs that generate both piRNAs and siRNAs but in different regions. (B) TEs tend to yield piRNAs. TEs listed here prefer to generate piRNAs not siRNAs. (C) TEs prefer to produce siRNAs. TEs presented here prefer to generate siRNAs not piRNAs. (D) Others.
(PDF)

Table S1 **The information of TE-miRNAs in silkworm genome.**
(PDF)

Table S2 **Results of TE-miRNA target prediction.**
(XLS)

Table S3 **TE-associated piRNAs and siRNAs in silkworm genome.**
(XLS)
