# Supplementary figures and images for: Transposable-Element Associated Small RNAs in Bombyx mori Genome
Source: PLoS One. 2012 May 8;7(5):e36599. doi: 10.1371/journal.pone.0036599 (PMC3359762; doi:10.1371/journal.pone.0036599)

Bm1770:

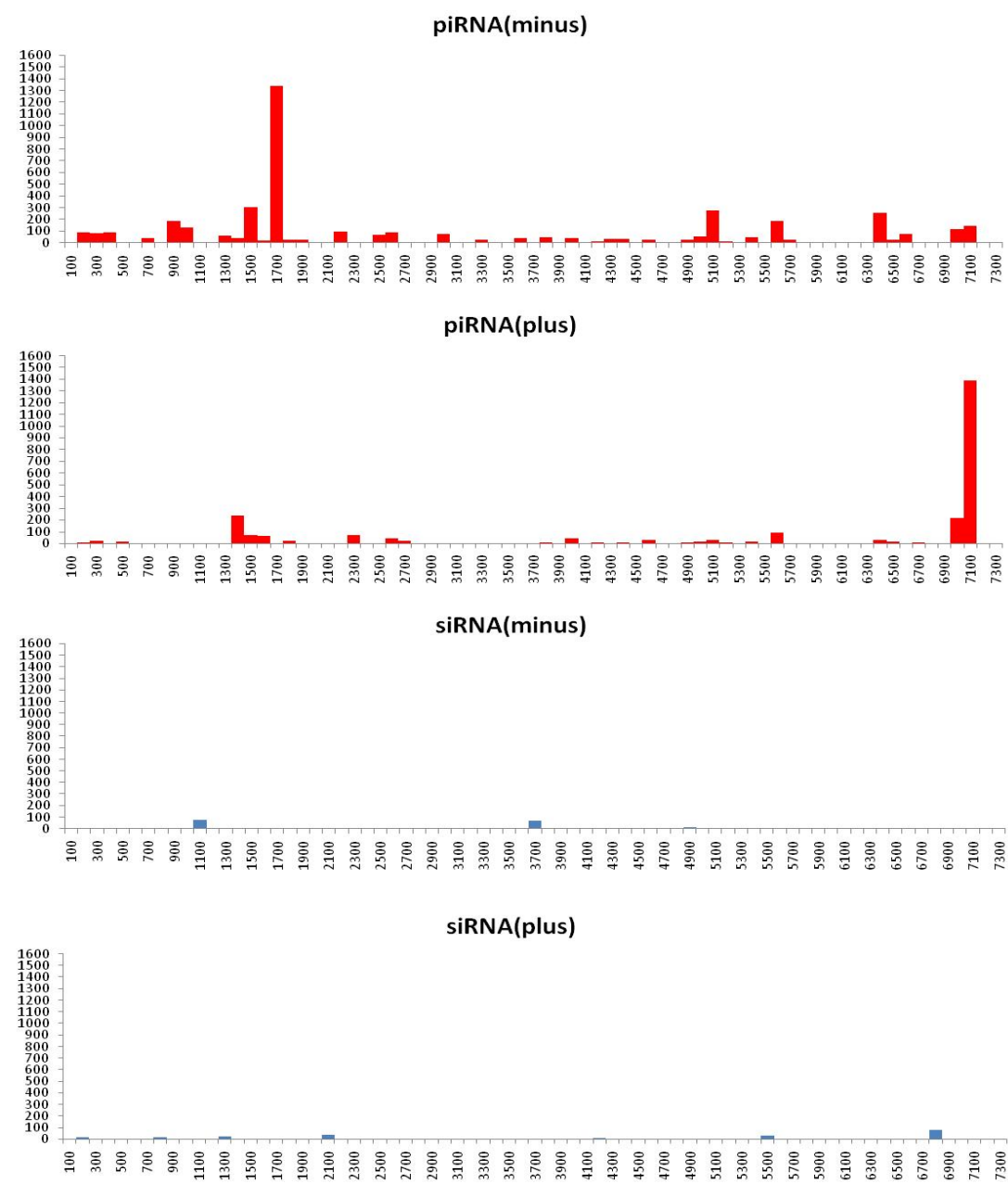

Bm219:

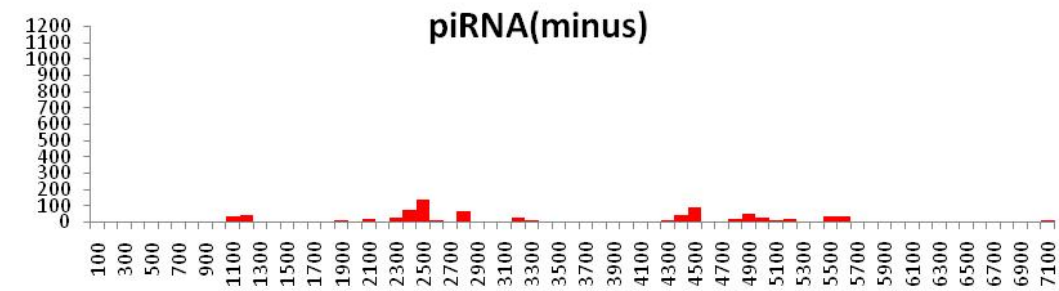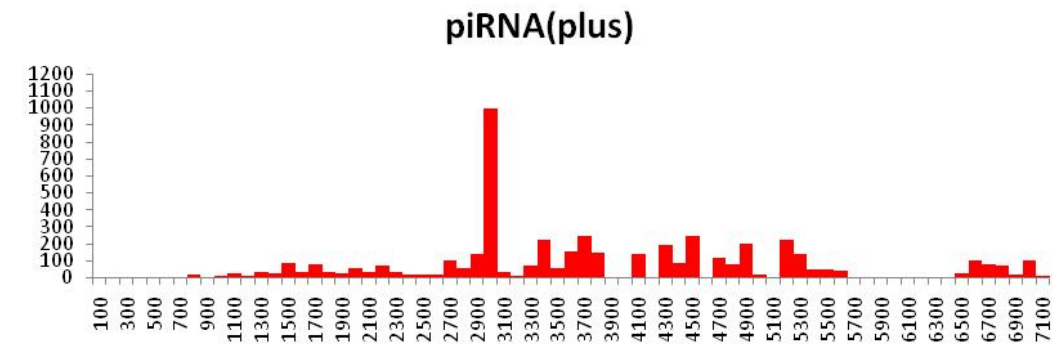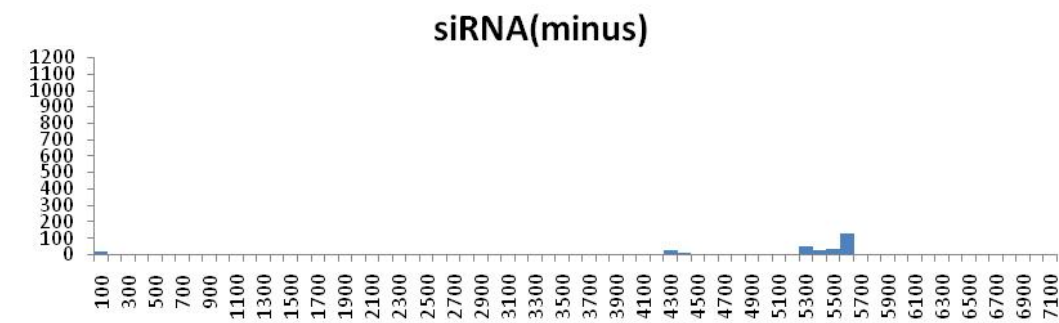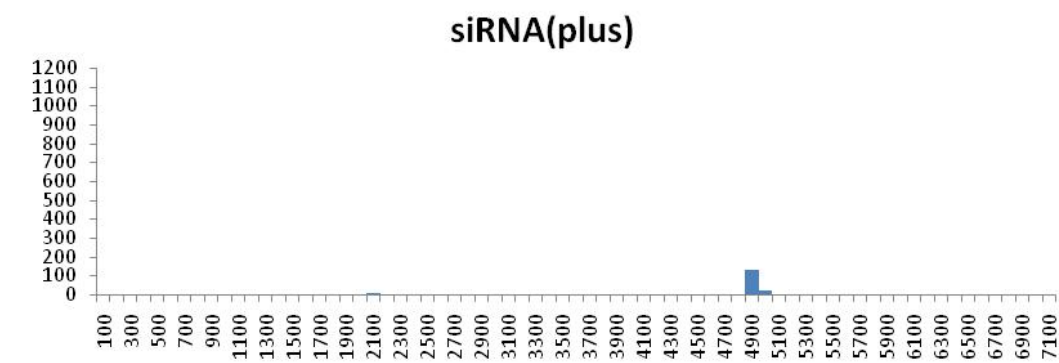

BMOPAORTA:

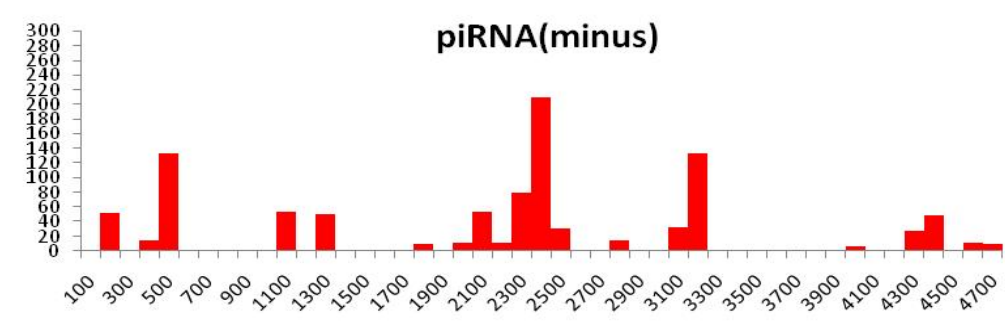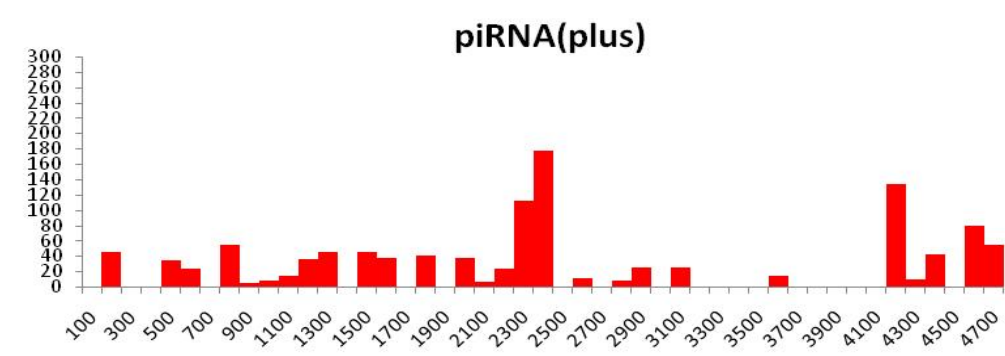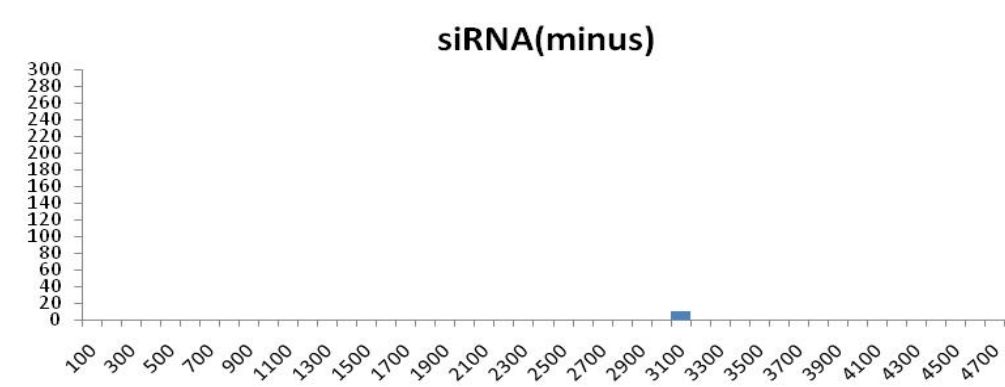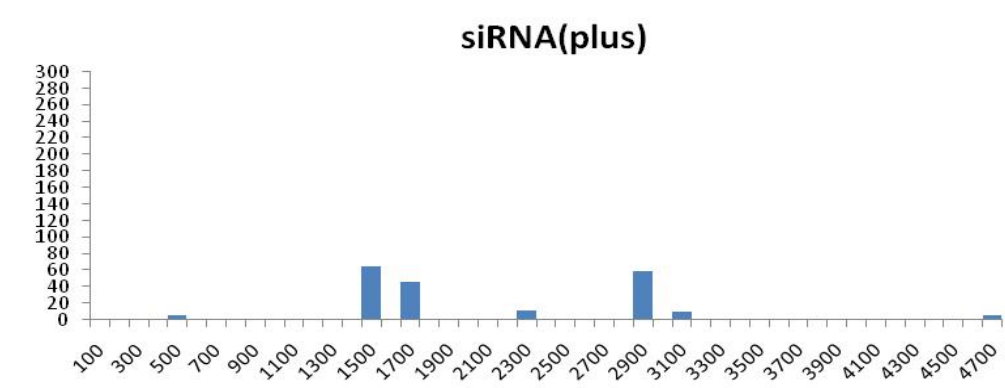

Takuya:

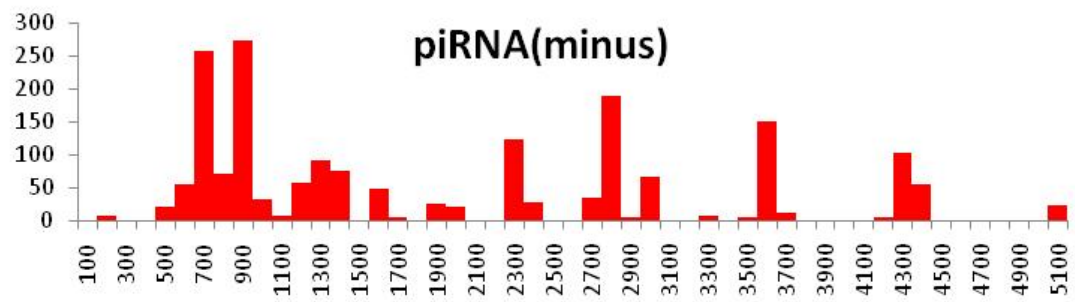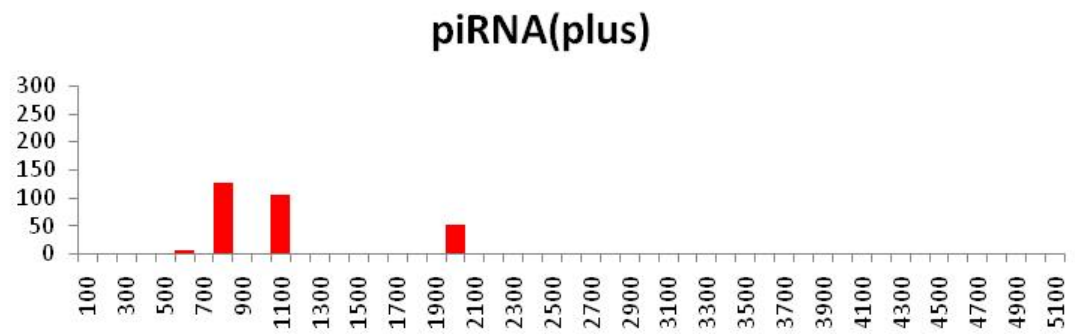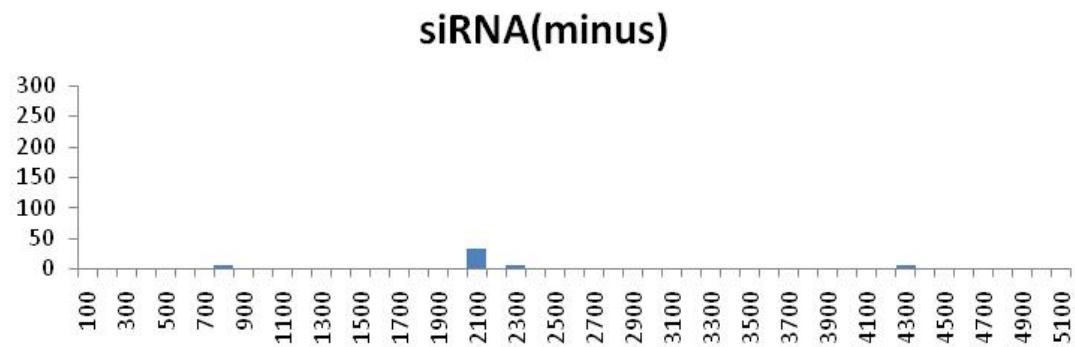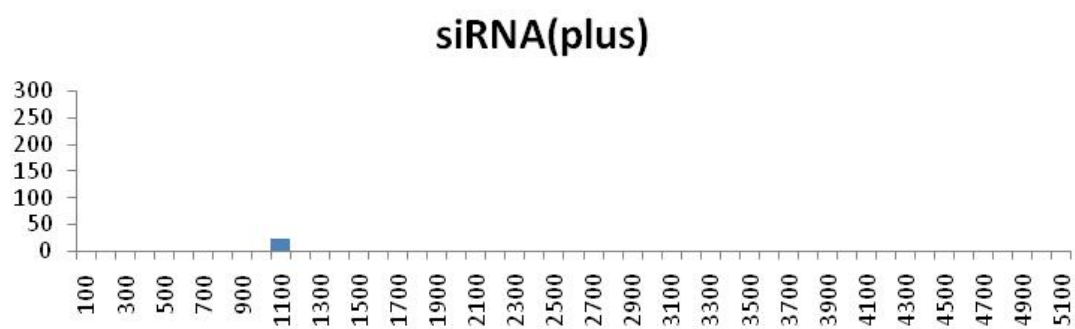

R2Bm:

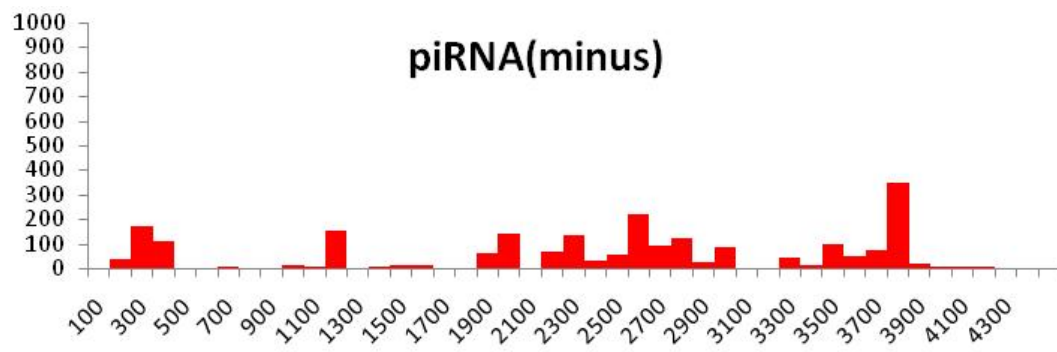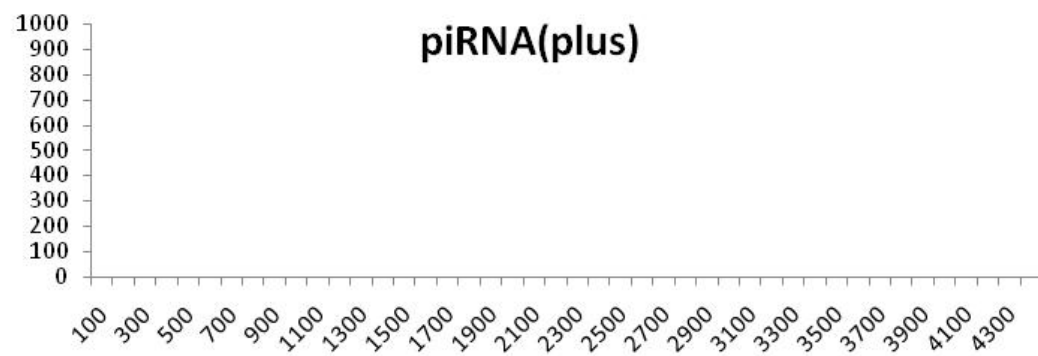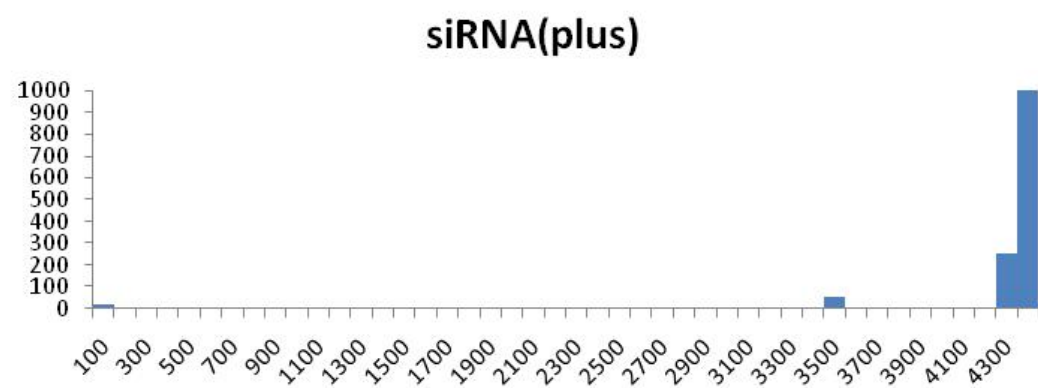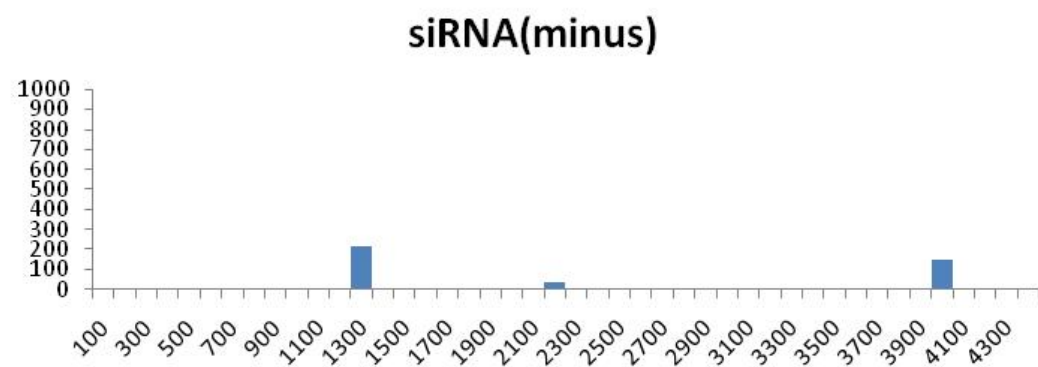

Supplement: Figure S1 — Representative silkworm TEs with mapped small RNAs. Silkworm TE-associated piRNAs and miRNAs are shown in red and blue, respectively. (PDF) [file pone.0036599.s001.pdf]

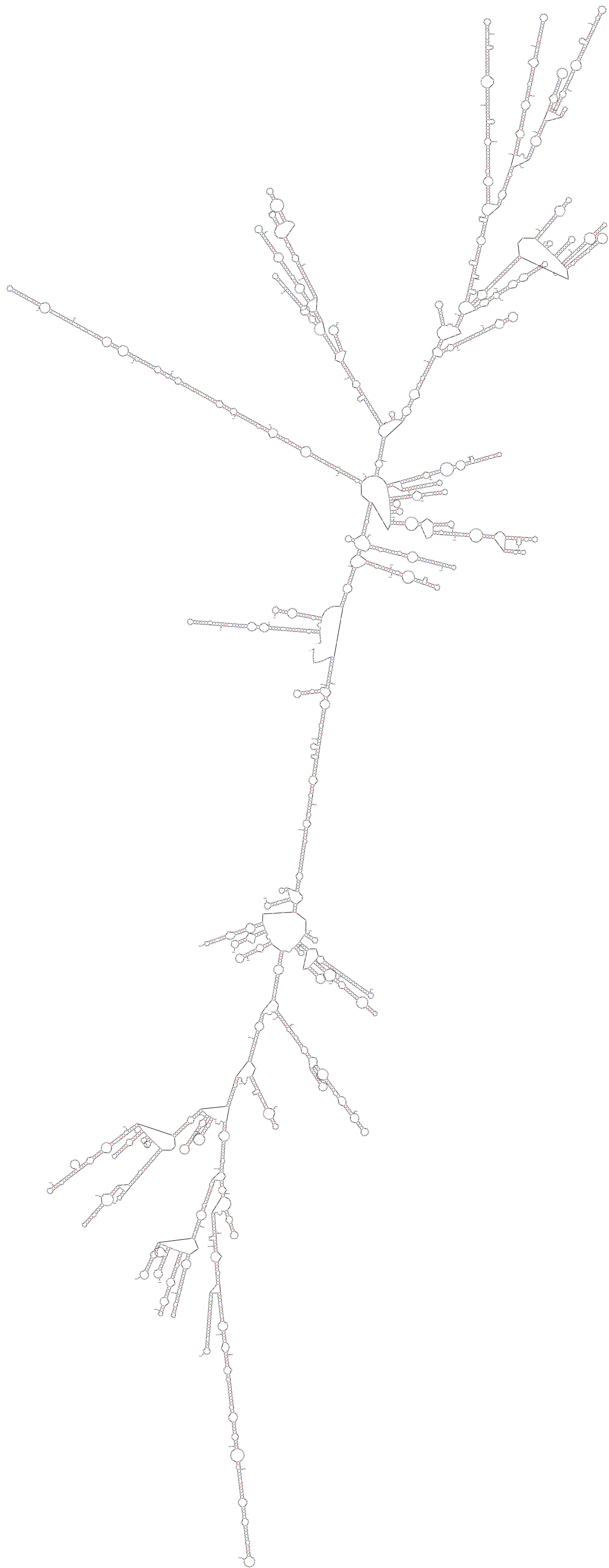

$dG = -1635.18$  *bm1645\_antisense* 30 centigrade

Supplement: Figure S2 — RNA folding results of the antisense strand of bm1645. (PDF) [file pone.0036599.s002.pdf]
