# Supplementary material for: Transposable-Element Associated Small RNAs in Bombyx mori Genome
Source: PLoS One. 2012 May 8;7(5):e36599. doi: 10.1371/journal.pone.0036599 (PMC3359762; doi:10.1371/journal.pone.0036599)

A. TEs with small RNA generating bias (fly).

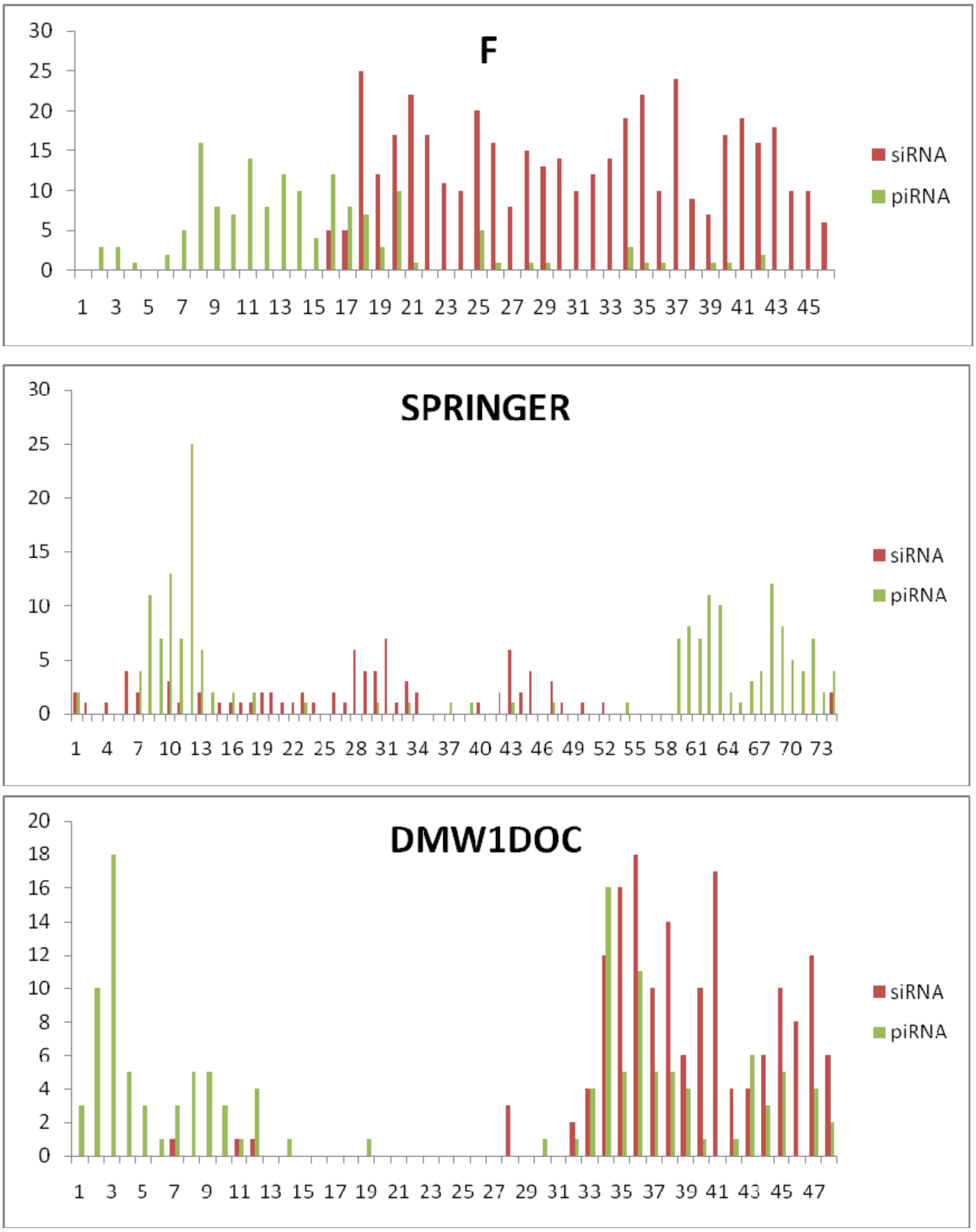

B. TEs tend to yield piRNAs (fly).

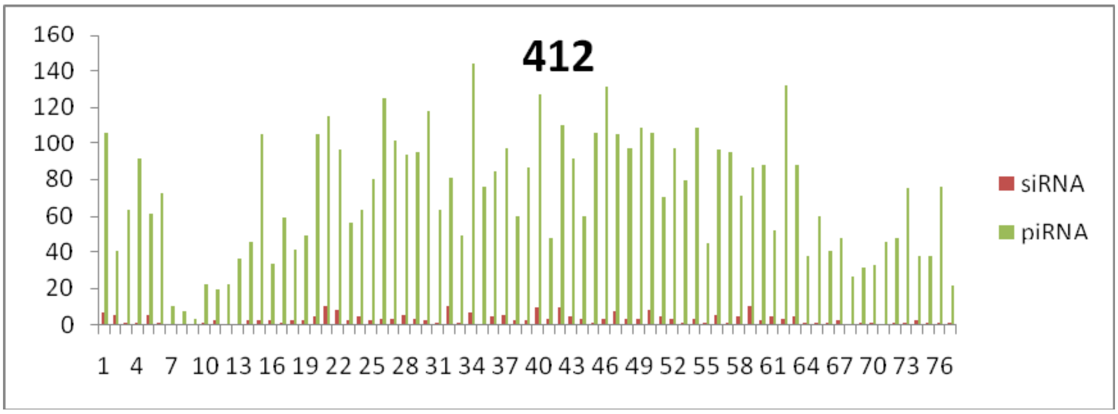
412
siRNA
piRNA

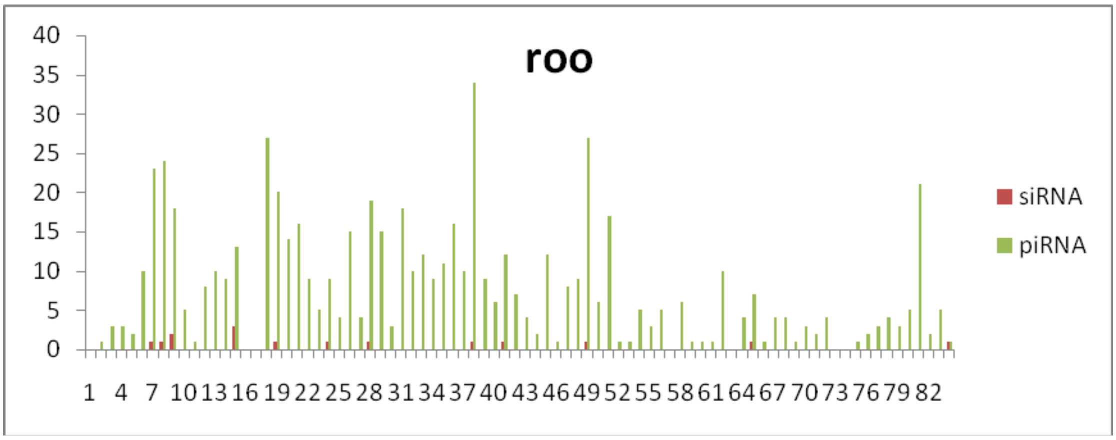
roo
siRNA
piRNA

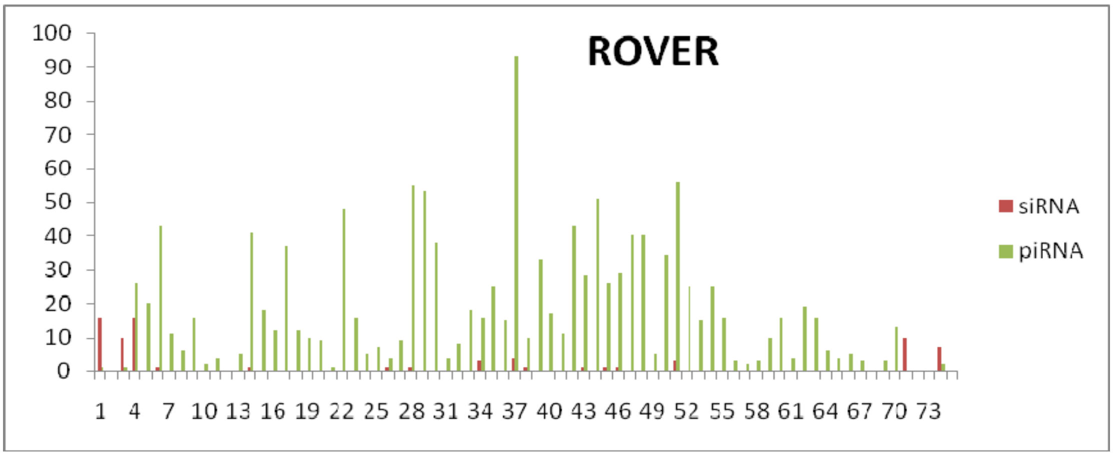
ROVER
siRNA
piRNA

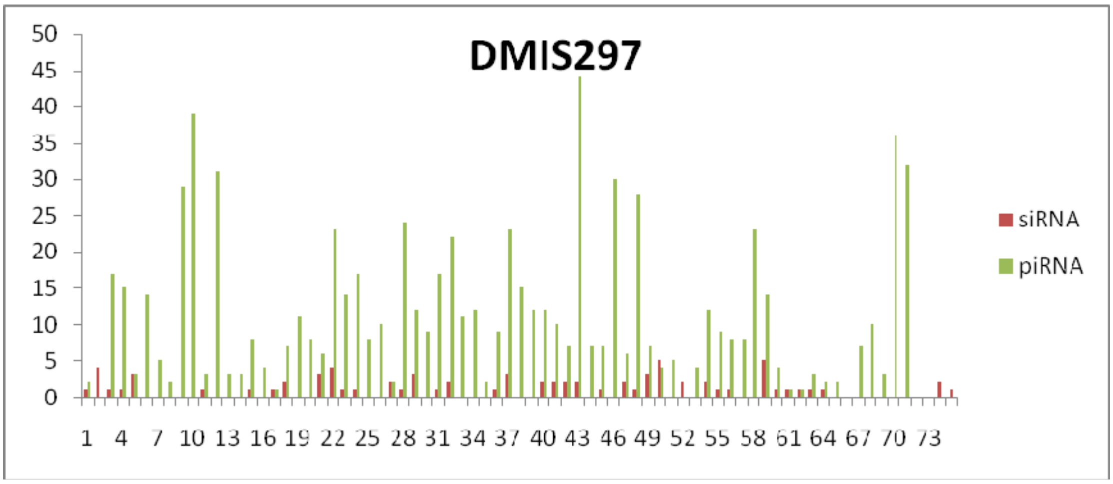
DMIS297
siRNA
piRNA

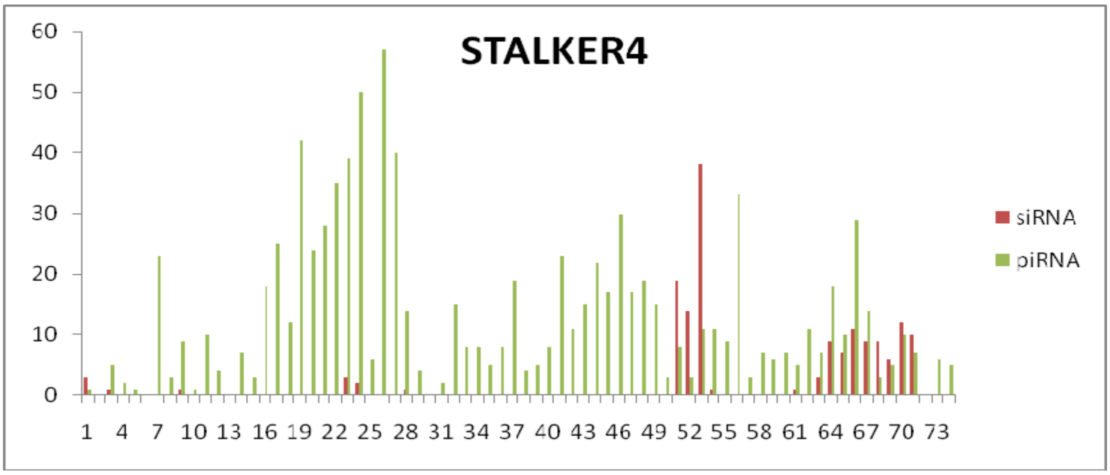
STALKER4
siRNA
piRNA

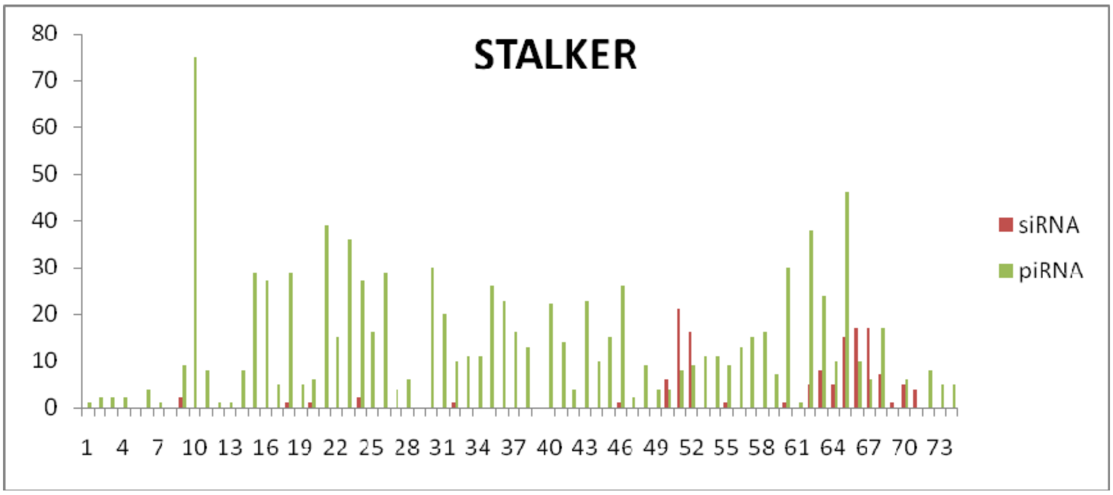
STALKER
siRNA
piRNA

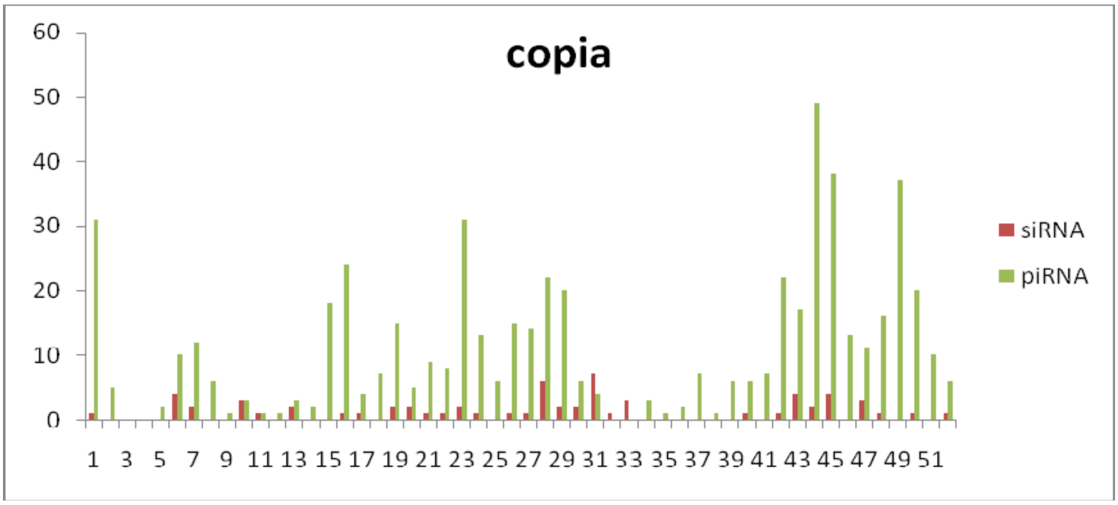
copia
siRNA
piRNA

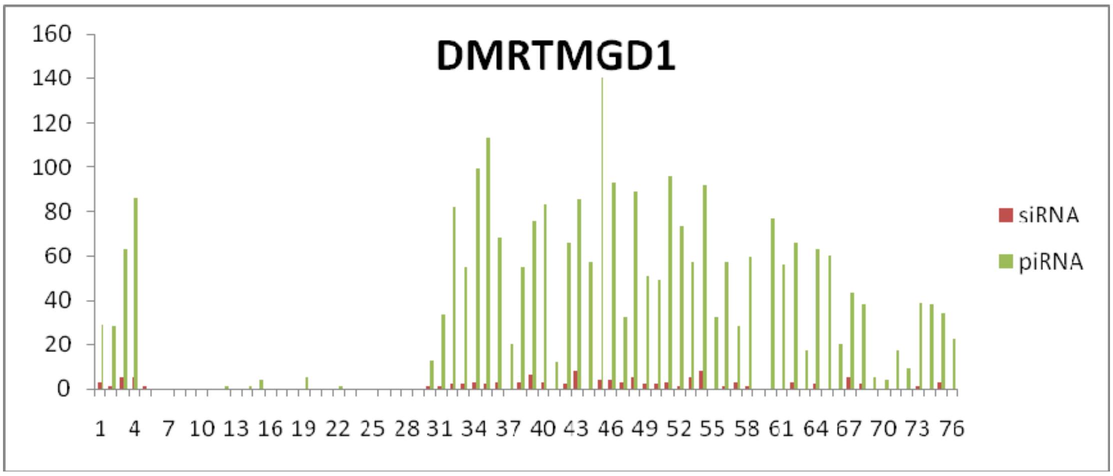

C. TEs prefer to produce siRNAs (fly).

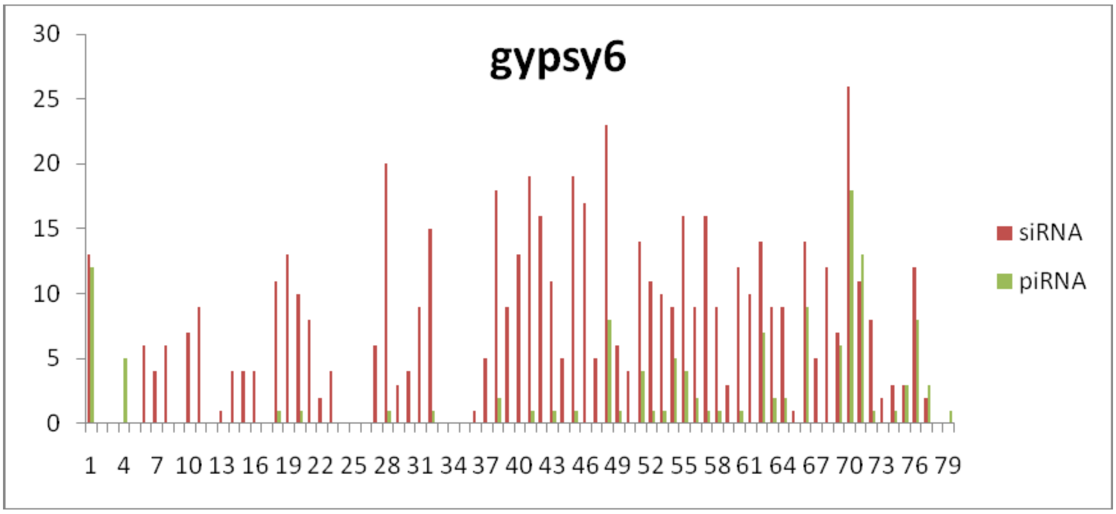

D.Others (fly).

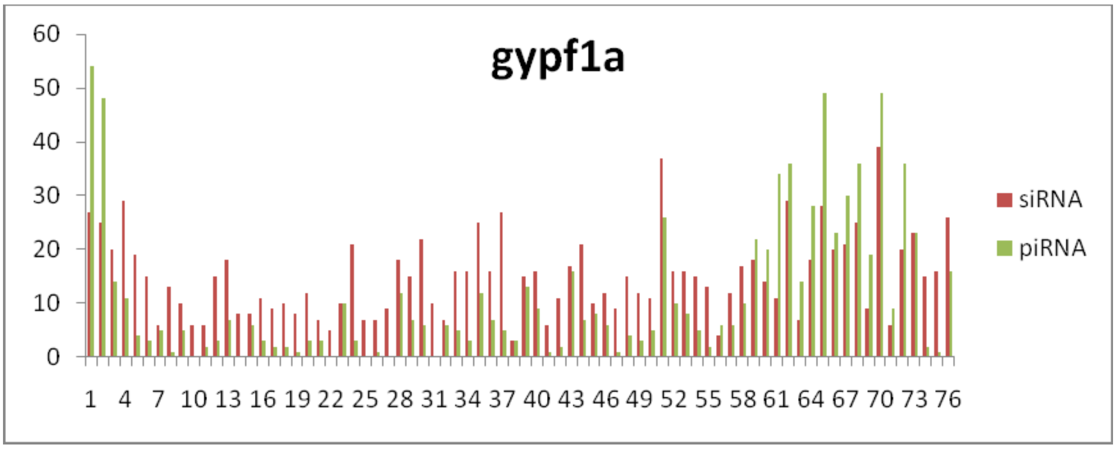

Supplement: Figure S3 — Fly TE mapped small RNAs. (A) TEs with small RNA generating bias. This part shows representative TEs that generate both piRNAs and siRNAs but in different regions. (B) TEs tend to yield piRNAs. TEs listed here prefer to generate piRNAs not siRNAs. (C) TEs prefer to produce siRNAs. TEs presented here prefer to generate siRNAs not piRNAs. (D) Others. (PDF) [file pone.0036599.s003.pdf]
