# Supplementary material for: Transposable-Element Associated Small RNAs in Bombyx mori Genome
Source: PLoS One. 2012 May 8;7(5):e36599. doi: 10.1371/journal.pone.0036599 (PMC3359762; doi:10.1371/journal.pone.0036599)
Supplement: Table S1 — The information of TE-miRNAs in silkworm genome. (PDF) [file pone.0036599.s004.pdf]

Table S1. The information of TE-miRNAs in silkworm genome.

TE-miRNAs	Sequence (5'-3')	Frequency	miRNA*	TE
bmo-miR-1923	TAATCGCGTACCGTTGCATAGCCGTGGC	-	no	bm_422
bmo-miR-2751	GTCTGGGCCGTGGAGCGTTT	-	no	Noguchi
bmo-miR-2814	TACAAATTCTGTGGTAGTAGGT	-	no	bm_236
bmo-miR-2819	TCAATGCCTGCTCTATCGGTTC	-	no	bm_1695
bmo-miR-2835	TCACACATTATGAGCTTTAGGA	-	no	bm_970
bmo-miR-2838	AATTCAGCAAACTCACGGGATAA	-	no	bm_903
bmo-miR-2842	TGAAGATCCTCGTACTGGTGGCGC	-	no	bm_1456
bmo-miR-2857	TGATGGGACACATACGGAACAT	-	no	bm_1556
bmo-miR-3305	TTACATATGGTTATTACGTCACTAG	-	no	bm_679
bmo-miR-3318	GGATTTTACATTTTTGCATGTTGC	-	no	bm_353, bm_191
bmo-miR-3329	GCATACAATAATTTATGACAGAT	-	no	bm_939
bmo-miR-3332	TCCTCGCCATGCCACCGCCGCTTCA	-	no	R2Bm
bmo-miR-3337	TCCATATCCGTGCTCGGACGCTT	-	no	bm_1770, bm_447
bmo-miR-3342	TGACCATGGTGACGAGTCGAGCCC	-	no	R2Bm, bm_1157
bmo-miR-3343	TCTCCATCGTTCCTGCACCGTAGC	-	no	bm_447
bmo-miR-3375	TGCTATCTTTGAAAAGTTTGGAATA	-	no	bm_219
bmo-miR-3379	AAATCTGACCGAAGACTACGA	-	no	bm_1645
bmo-miR-3384	TGTCTGTTTGTGCGCTGGTAGAAGA	-	no	BMOPAORTA
bmo-miR-3333	CCGTTCGAGAAGCAAGACAAAGTG	-	no	Moriya
bmo-miR-3304a	TAAACAGCTTGGAATATTTACAG	-	no	Benkei
bmo-miR-3314	ACGCAGCAACAGCGGCCGCCTCC	-	no	TREST-W
bmo-miR-3389	TCGTAGCCGATGTTCCACAGCAG	-	no	TREST-W
TE-miRNA-1	TGTAAGGAGACATGAGAGGTG	18	yes	bm_1645
TE-miRNA-2	TGGCATTGTAAGGAGACATGA	104	yes	bm_1645
TE-miRNA-3	CTGCCTGAGCGTTCGAGTTCCAT	619	yes	bm_1645
TE-miRNA-4	GGCGGGGAGTTTGACTGGGGC	122	yes	bm_1645
TE-miRNA-5	CCAGGCGGGGAGTTTGACTGG	85	yes	bm_1645
TE-miRNA-6	GGGCAGCTTCCGGGAAACCAA	237	yes	bm_1645
TE-miRNA-7	AGCCCAGCACTGAATCCCGTTGTT	25	yes	bm_1645
TE-miRNA-8	ATTCAGGTGAGGGATGTACGTG	16	yes	bm_1645
TE-miRNA-9	GATGCGGCCGGTGCCGGGTCT	21	yes	bm_1645
TE-miRNA-10	CGCGTCTGTTGTCGCAGCCGTGC	20	yes	bm_1645
TE-miRNA-11	CTGTATATATTTAATATGACACT	135	no	bm_1645
TE-miRNA-12	TTGGTATCATTAAGCCGTAACG	5	no	bm_1645
TE-miRNA-13	TCACCAGGCCCGGACACCGGAA	59	no	bm_1645
TE-miRNA-14	TTCCTAGCCGTAAGGTCGTGTC	348	no	bm_1645
TE-miRNA-15	GAATGATTTAGTGAGGTCTTCG	52	no	bm_1645
TE-miRNA-16	GGCTTGGGAGAATCAGCGGGGA	229	no	bm_1645
TE-miRNA-17	ATGGTTCGGAACGCGAAGAGCAC	28	no	bm_1645

TE-miRNA-18	AGAACACGTTCGGACCGCGTAT	426	no	bm_1645
TE-miRNA-19	GTGCGAGTCATTGAGTTTTATAA	18	no	bm_1645
TE-miRNA-20	CTTGGAGCCTCCGTCGGTGCA	88	no	bm_1645
TE-miRNA-21	TTTTCGCACTCCCGAGGCGTCT	15	no	bm_1645
TE-miRNA-22	ATCCGCTAAGGAGTGTGCAACGA	178	no	bm_1645
TE-miRNA-23	CATTGTAAGGAGACATGAGAG	24	no	bm_1645
TE-miRNA-24	CGGTGTTTCGTTCCAAGCGTGCAG	730	no	bm_1645
TE-miRNA-25	GTTTGTTTGAACGTCTGCGATGA	32	no	bm_1645
TE-miRNA-26	ATGTGTGCGAGTCATTGAGTT	77	no	bm_1645
TE-miRNA-27	CCGAGGCGTCTCGTTTCCAATCAG	16	no	bm_1645
TE-miRNA-28	AGCCCGCTTGGAGCCTCCGTCG	9	no	bm_1645
TE-miRNA-29	TCCGGCCTTCCGCGGATCTTCC	7	no	bm_1645
TE-miRNA-30	CCCTGTTGAGCTTGACTCTAGTCT	5	no	bm_1645
TE-miRNA-31	TCCCTTGTTGCTCCTGGTGAT	8	no	bm_447
TE-miRNA-32	TATTAGCACGGGTGATGCCAGCC	8	no	bm_1770
TE-miRNA-33	ATCTTCCTAGCCGTAAGGTCGT	5	no	bm_1645
TE-miRNA-34	CGGTATCATTAAACTAATCACGCA	7207	no	bm_1645
TE-miRNA-35	CCGGCCTTCCGCGGATCTTCCT	156	no	bm_1645
TE-miRNA-36	GGTTGGGCGGAAGCGGTGCGC	33	no	bm_1645
TE-miRNA-37	ACCGCGTATCGTTCCGATCCA	1336	no	bm_1645
TE-miRNA-38	AGCCTCCGTCGGTGCAGATCT	226	no	bm_1645
TE-miRNA-39	CAAACTTGATCATTTAGAGGA	378	no	bm_1645
TE-miRNA-40	CAGATCAGGGAGGATCACCCGC	665	no	bm_1645
TE-miRNA-41	TTGAACGTCTGCGATGATACA	657	no	bm_1645
TE-miRNA-42	CCGATTGAATGATTTAGTGAG	511	no	bm_1645
TE-miRNA-43	ATGGCTCGGCGGGCAGCTTCCGG	119	no	bm_1645
TE-miRNA-44	CCAAGTTCGTCTTGAACGGGGC	651	no	bm_1645
TE-miRNA-45	AGCCGGAGATCTGATGACGGTGC	980	no	bm_1645
TE-miRNA-46	CCGCAGCAGGTCTCCAAGGTGAA	158	no	bm_1645
TE-miRNA-47	ACGCGAGACGCGACGTCGAAGCC	1256	no	bm_1645
TE-miRNA-48	GGCGCACGGTGTTTCGTTCCA	556	no	bm_1645
TE-miRNA-49	AGCATGTGTGCGAGTCATTGA	633	no	bm_1645
TE-miRNA-50	GGCAAATTGGATCCGTAACTTC	84	no	bm_1645
TE-miRNA-51	GCACAATTGATCTCACATCCAA	21	no	bm_1266
TE-miRNA-52	CTTACTCGGTTGGGCGGAAGCG	204	no	bm_1645
TE-miRNA-53	CGTTGGTCGCTCATGACAGCA	201	no	bm_1645
TE-miRNA-54	AGCACTGAATCCCGTTGTTTTA	85	no	bm_1645
TE-miRNA-55	GTCTGGCATTGTAAGGAGACA	29	no	bm_1645
TE-miRNA-56	TCATTACTTACTCGGTTGGGCG	197	no	bm_1645
TE-miRNA-57	GCGAGGCCCTGGAGGACTGAC	48	no	bm_1645
TE-miRNA-58	CTCGAATGAACGAACGGAGAG	1737	no	bm_1645
TE-miRNA-59	GGTCTGGTCGATGTTCGTGCG	240	no	bm_1645
TE-miRNA-60	GCGTATCGTTCCGATCCACGC	282	no	bm_1645
TE-miRNA-61	CCGAAGTTTCCCTCAGGATAG	1032	no	bm_1645

TE-miRNA-62	GTCGCTACTACCGATTGAATGA	349	no	bm_1645
TE-miRNA-63	TCTAATAGCGTCTTGGCGGAGAA	46	no	bm_375
TE-miRNA-64	GTAGCTGGTTCCGTCCGAAGTT	86	no	bm_1645
TE-miRNA-65	GGACCGGGGCGTGTCGGGTTTG	127	no	bm_1645
TE-miRNA-66	AAGTTTCCCTCAGGATAGCTGG	181	no	bm_1645
TE-miRNA-67	GGGGTAAACCTGCGAAACTCGA	365	no	bm_1645
TE-miRNA-68	TCGCAGCCGTGCAGTCTCGGA	440	no	bm_1645
TE-miRNA-69	CGTTTTCTCTCGGGCGTACGT	176	no	bm_1645
TE-miRNA-70	CGCGTGAACAGTAGTTGCTCGC	114	no	bm_1645
TE-miRNA-71	ATGGCGAACTGTATCTTTCCCG	42	no	AB018558,BMC1, BMC1-Abe,Kabuki
TE-miRNA-72	ACAGTTTCGGGCACTCGCAGGACC	864	no	bm_1645
TE-miRNA-73	CTGACGTGGAGAAGGGTTTCGC	55	no	bm_1645
TE-miRNA-74	GGTGAGGGATGTACGTGGAGA	143	no	bm_1645
TE-miRNA-75	TCCGCGGATCTTCCTAGCCGT	328	no	bm_1645
TE-miRNA-76	TGACCAAACTTGATCATTTAG	105	no	bm_1645
TE-miRNA-77	CCTGTTGAGCTTGACTCTAGTC	143	no	bm_1645
TE-miRNA-78	CTCCGTCGGTGCAGATCTTGGT	88	no	bm_1645
TE-miRNA-79	GTTGTCGCAGCCGTGCAGTCTC	132	no	bm_1645
TE-miRNA-80	CGTTTTCCCGTAGAGGGTGCCA	364	no	bm_1645
TE-miRNA-81	AGTCTGGCATTGTAAGGAGACAT	7	no	bm_1645
TE-miRNA-82	AGGGTTTCGCGTGAACAGTAGT	42	no	bm_1645
TE-miRNA-83	GTTGGCATCTATTTTCAATGC	18	no	AB018558,BMC1, BMC1-Abe,Kabuki
TE-miRNA-84	AATGGGTGAGAACTCCGGCTTACT	46	no	bm_1645
TE-miRNA-85	TAGCAAATACTCCAGCGAGGC	53	no	bm_1645
TE-miRNA-86	CGAGTAGGACGTGCGCGACGG	89	no	bm_1645
TE-miRNA-87	TATGTCGATGTGGCGTGTTTT	5	no	bm_1645
TE-miRNA-88	GTGCGTTCGTTCTCCGTCGGCACG	96	no	bm_1645
TE-miRNA-89	GCGACCACCAGAACGCACCCCCA	7	no	bm_8
TE-miRNA-90	CTGGGGCGGTACATCTGTCAAA	59	no	bm_1645
TE-miRNA-91	CTTACGTTTCGGACTGGATCC	143	no	bm_1645
TE-miRNA-92	CTTTAAATGGGTGAGAACTCCGG	288	no	bm_1645
TE-miRNA-93	AGCCGTAAGGTCGTGTCGGTTTC	63	no	bm_1645
TE-miRNA-94	CGTTTCGGACTGGATCCGGACCCG	454	no	bm_1645
TE-miRNA-95	GGACTGTGGTGGATTGGCACA	6	no	AB018558,BMC1, BMC1-Abe,Kabuki, Supermite-BMC1
TE-miRNA-96	TTCGTTCCAAGCGTGCAGAGTG	64	no	bm_1645
TE-miRNA-97	GAAACTCCTTCGTGCTGGGGA	103	no	bm_1645
TE-miRNA-98	GCGATCGGCACGATTCTGTAC	56	no	bm_1645
TE-miRNA-99	TTCGTTCTCCGTCGGCACGGTAC	158	no	bm_1645
TE-miRNA-100	GGGAAGTCGGCAAATTGGATCC	37	no	bm_1645
TE-miRNA-101	TCCAAGCGTGCAGAGTGGTGA	43	no	bm_1645

TE-miRNA-102	TCTAGTCTGGCATTGTAAGGAGA	218	no	bm_1645
TE-miRNA-103	GAACGGGCTTGGGAGAATCAGC	67	no	bm_1645
TE-miRNA-104	GGTAGTAGCAAATACTCCAGC	137	no	bm_1645
TE-miRNA-105	TAGGACGTGCGCGACGGAGAG	45	no	bm_1645
TE-miRNA-106	TTGATCATTTAGAGGAAGTAA	35	no	bm_1645
TE-miRNA-107	AAGAACATTATGTCGTACGTGA	28	no	Noguchi,bm_219
TE-miRNA-108	AGGCGTCTCGTTTCCAATCAGTGA	122	no	bm_1645
TE-miRNA-109	CGCACTCCCGAGGCGTCTCGT	96	no	bm_1645
TE-miRNA-110	TTGACATTACCGCGCTAGTCA	5	no	bm_1770
TE-miRNA-111	GGTGTCCGGATACTCTCTGCGGAC	47	no	bm_1645
TE-miRNA-112	TATTTAATATGACACTCGCGA	22	no	bm_1645
TE-miRNA-113	GTTTCATTACTTACTCGGTTG	53	no	bm_1645
TE-miRNA-114	CTACTACCGATTGAATGATTTA	50	no	bm_1645
TE-miRNA-115	GTTCCGTCCGAAGTTTCCCTC	370	no	bm_1645
TE-miRNA-116	GATCACGCCGTACGAGCGTTTT	8	no	bm_1645
TE-miRNA-117	GCACGGTGTTTCGTTCCAAGCG	58	no	bm_1645
TE-miRNA-118	AACGCCCTTTGAGCGAAAGGGA	77	no	bm_1645
TE-miRNA-119	CTGCCTTAGTGCGGACGCGAGTG	14	no	bm_1645
TE-miRNA-120	GTGCGCGATCGGCACGATTCT	64	no	bm_1645
TE-miRNA-121	GTTCCGCGTTACGCGTTCGTACT	14	no	bm_1645
TE-miRNA-122	CGTCGAAACGTCGCGTACACGTCC	8	no	bm_1645
TE-miRNA-123	GGAAGCGGTGCGCGGTCGATAAT	51	no	bm_1645
TE-miRNA-124	TGCGATCTGAAGTATCTATCCA	11	no	bm_1645
TE-miRNA-125	TTCCAAGCGTGCAGAGTGGTG	39	no	bm_1645
TE-miRNA-126	CCGCTGAAACTCCTTCGTGCT	230	no	bm_1645
TE-miRNA-127	GATTTTTCGCTTGCACACACAT	6	no	bm_1645
TE-miRNA-128	GCCTGTGTTCGTGGTACATCTGC	5	no	bm_26
TE-miRNA-129	CGTGCGTTATGTTCGTTTTAGC	100	no	bm_1645
TE-miRNA-130	TGTTGAGCTTGACTCTAGTCT	14	no	bm_1645
TE-miRNA-131	CTGGTTCCGTCCGAAGTTTCC	22	no	bm_1645
TE-miRNA-132	ATTGAATGATTTAGTGAGGTC	19	no	bm_1645
TE-miRNA-133	GACCGAAGACTACGATTACGA	9	no	bm_1645
TE-miRNA-134	TCTGAAGTATCTATCCATCTCTCA	12	no	bm_1645
TE-miRNA-135	CGCCGTAACACCGTGTGAGAC	11	no	bm_1645
TE-miRNA-136	CGAGACCACGCCTCCAGCACGGAC	8	no	bm_58,TREST-W
TE-miRNA-137	GTTTCGGGCACTCGCAGGACCCGT	10	no	bm_1645
TE-miRNA-138	TACTGTTGCTTCATGGGGTCAA	11	no	bm_903
TE-miRNA-139	TTTCTCTCGGGCGTACGTTTAC	11	no	bm_1645
TE-miRNA-140	TCGACGGTGTATCGCGTCCCGA	6	no	TREST-W
TE-miRNA-141	AACCCGCTGAAACTCCTTCGT	15	no	bm_1645
TE-miRNA-142	ATTACGAATCGCGTTTCGCTA	7	no	bm_1645
TE-miRNA-143	CAAACCTAGAAGACACCGAACATA	6	no	bm_1027
TE-miRNA-144	CCGTCCGAAGTTTCCCTCAGGA	51	no	bm_1645
TE-miRNA-145	CCATCTCTCATATATATCTTGTC	20	no	bm_1645

TE-miRNA-146	GACGTGCGCGACGGAGAGCGCAG	5	no	bm_1645
TE-miRNA-147	ATGACAGCAGGACGGTGGCCAT	51	no	bm_1645
TE-miRNA-148	TCGTACAGTGCCTCTATCGCTAGA	37	no	Pakurin
TE-miRNA-149	TGGATCATAGGCAGCGGCGTCTT	23	no	R2Bm
TE-miRNA-150	CATTACATTAAACTATGAACTA	24	no	Takuya
TE-miRNA-151	CGTGACTTCATAGAGCCTGGC	23	no	BmpiggyBac
TE-miRNA-152	CCTCGTTCGTGCTGCGATAGA	5	no	Bmmar6
TE-miRNA-153	GTCTCACCTTTCGTCTGGTGCAG	7	no	HOPEBm2
TE-miRNA-154	ACGCCACCGGTAATCGGCAGCGAG	8	no	TREST-W
TE-miRNA-155	TCTCGAACGGGACGGTACCTGTAT	5	no	Moriya
TE-miRNA-156	AACGCCTTCAACACCCTGCCCT	7	no	TREST-W
TE-miRNA-157	CATGTCACACGAAATGCGTTATG	5	no	Minichikuri
TE-miRNA-158	TAGCTCAAGCTCCTCGTGCACC	5	no	Pakurin
TE-miRNA-159	CTGTATGAGCGTCTGCTCTACAAA	9	no	BMC1,BMC1-Abe, Kabuki,L1Bm
TE-miRNA-160	TCCAACAGTCCCCCCTCCCAG	7	no	Pakurin
